# The place of the public under COVID‐19

**DOI:** 10.1111/cag.12769

**Published:** 2022-05-27

**Authors:** Vanessa Mathews

**Affiliations:** ^1^ Department of Geography & Environmental Studies University of Regina

**Keywords:** public space, inequity, COVID‐19, urban centres, Canada, espace public, iniquité, COVID‐19, centres urbains, Canada

## Abstract

Over the past several decades, scholars have lamented the erosion of “true” public space through the rise of semi‐public and private spaces where access is determined, and usage is increasingly regulated (e.g., through curfews and restrictions on use). Writing in the midst of the sixth wave of COVID‐19, the importance of inclusive, open spaces in Canadian urban centres is evident: these are spaces that allow for movement and participation (ideally) across axes of identity and difference. Yet, public spaces are also bound up with the heightened regulation of bodies to supress contagion through social distancing and restrictions on use. While some bodies are in place (and take up space) in public in this context, other bodies face limitations pertaining to discrimination, surveillance, health, and access. This viewpoint highlights the importance of retaining and producing inclusive public spaces and the need to think critically about the urban experience under COVID‐19.

## Key Messages

- During COVID‐19, some streets were transformed into active transportation corridors, but access to these sites was uneven.
- Rising hate crimes and discrimination, alongside a law enforcement approach to public health, exacerbated unequal access, belonging, and safety for marginalized groups in public spaces.
- Planning practices must be particularly monitored across urban centres during the crisis, to ensure compliance with long‐term plans emphasizing compact development and shared resources.

## L'espace public sous l'emprise de la COVID‐19

*Au cours des dernières décennies, les chercheurs ont déploré l'érosion d'un « véritable » espace public par la montée des espaces semi‐publics et privés dont l'accès est déterminé et l'usage de plus en plus réglementé (par exemple, par des couvre‐feux et des restrictions d'utilisation). Au milieu de la sixième vague de COVID‐19, l'importance des espaces ouverts et inclusifs dans les centres urbains canadiens paraît évidente: il s'agit d'espaces qui permettent le mouvement et la participation (idéalement) sous l'angle de l'identité et de la différence. Pourtant, les espaces publics sont également liés à la régulation accrue des individus afin de supprimer la contagion par la distance physique et des règles d'usage contraignantes. Si certains individus sont présents (et occupent l'espace) en public dans ce contexte, d'autres individus sont confrontés à des limitations en matière de discrimination, de surveillance, de santé et d'accès. Ce point de vue souligne l'importance de conserver et d'établir des espaces publics inclusifs et la nécessité de réfléchir de manière critique à l'expérience urbaine dans le cadre de la COVID‐19*.

## 1 Introduction

Public space has long been regarded as a site of contradiction and ambiguity, bound up in a wrestle between freedom/exploration and restriction/regulation depending on the bodies that produce, reproduce, and control its functions. True public space, by definition, is designed to be inclusive space that belongs to everyone under the regulation of laws and norms. In practice, public space has always excluded particular bodies and privileged others (Mitchell 1995, 2017). To what extent can public space belong to everyone during a global pandemic? How are spaces being remade and reproduced to meet new demands? Are inequities within public space extenuated during the crisis? In this viewpoint, I highlight the changing nature of urban public space in the Canadian context under COVID‐19. My focus is on the current crisis: an evolving and mutating set of restrictions, regulations, and responses.

I argue that the COVID‐19 pandemic has deepened inequities associated with public space. First, the pandemic demonstrated the potential to transform streets and parking spots from their function for vehicular movement, into public spaces for active transportation and social exchange. However, new active street programs in Canadian municipalities during COVID‐19 were concentrated in areas with lower rates of visible minority populations and fewer children, highlighting uneven access (Fischer and Winters 2021). Second, during the pandemic, participation and belonging in public space for racialized and marginalized populations were limited by racism and unequal law enforcement, factors which have been exacerbated over the past two years, despite the importance placed on access to public space for mental and physical health and for social distancing. Data emerging from Statistics Canada alongside that from numerous organizations and associations, highlight increased incidents of anti‐Asian racism and xenophobia in public and semi‐public spaces since 2020. Third, a demand for decentralized private space (e.g., larger lots and cottages) by those able to afford it has placed pressure on intensification planning. Policy and planning actions across Canadian urban centres must be particularly monitored during the current crisis to ensure that actions are not departing from long‐term development goals that prioritize shared resources.

## 2 Reconstituting public spaces

It has become clear over the past 24 months that our public spaces are limited. They are limited in design and access. Urban geographer and artist Daniel Rotsztain made headlines after wearing a 2‐metre social distancing machine through the busy streets of Toronto to advocate for street closure of major thoroughfares to keep the public safe (Rocca 2020). Pilot projects across urban centres in Canada have illustrated a capacity to produce new spaces for public use, including reorienting spaces for vehicular movement (e.g., streets and parking lots) into sites of active transportation for cyclists and pedestrians (Combs and Pardo 2021). Vehicular traffic as well as public transit ridership was down across the country making these temporary modifications to space possible. By September 2021, 29 Canadian urban centres (from small population centres to large urban centres) had implemented changes to their streets to encourage active mobility (Combs and Pardo 2021). Montreal's plan not only closed down streets to increase the number of public spaces, it considered the need for these spaces in its high density neighbourhoods to alleviate pressure on the park system (MacFarlane 2020). This consideration of access to private space was not shared across municipalities in planning for active transportation routes. The concentration of active transportation corridors across Canadian municipalities in areas that lacked diversity is striking (see Fischer and Winters 2021).

Streets are critical public spaces, associated with counter movements and multifunctional activities. Over time, the primary function of these spaces for vehicular movement has largely defined their use. The reclamation of street space for non‐vehicular purposes transforms the meaning of these sites and disrupts historic planning practices that have oriented our cities around automobile networks. Most municipalities have been clear that these are temporary measures and there should be no assumption of their continuation long‐term. At the same time that public spaces are being reoriented for active transportation across Canadian cities, private usage is encroaching into public spaces as regulations surrounding street retail, patios. and terraces are relaxed, spilling over onto sidewalks (Combs and Pardo 2020; March and Lehrer 2021).

Canada's urban centres have worked to manage contagion by opening and closing down public spaces (e.g., parks, playgrounds, streets) at various points to control local transmission rates. The closure of parks and spaces of play, especially during the beginning stages of the pandemic when contagion risks outdoors were relatively unknown, had an uneven effect on low‐income, racialized populations (Rodriguez 2021). These are populations that generally have less access to public space amenities as well as less access to private space, both indoor and outdoor. Whether these shifting public spaces will permanently transform urban planning and design in the future remains uncertain (Honey‐Rosés et al. 2020). Municipalities should focus on public spaces, both permanent and flexible, in low‐income/high‐density areas to account for uneven access to private/public space.

## 3 Reconstituting “the public”

During COVID‐19, temporary arrangements of time and mobility have transformed public space and who is seen within it. Modifications to public space for active transportation are always already structured by race, class, gender, and sexuality. In other words, these spaces are not experienced, accessed, or distributed equally. I want to briefly sketch out two distinct groups—racialized populations and the homeless—to highlight how fear, access, and differential systems of belonging in public space have become exacerbated during COVID‐19.

Anti‐Asian racism and xenophobia have intensified since March 2020 across Canadian cities. Police reported hate crimes (e.g., physical and verbal attacks, racial slurs, and COVID‐19 accusations motivated by hatred directed to a particular race or ethnicity) saw an 80% increase in 2020 compared to the year prior (Statistics Canada 2022), with crimes directed at East or Southeast Asian people up by 301% (*National Post* 2022). Incidents of hate crimes during the pandemic, reported anonymously on a COVID racism website developed by the Chinese Canadian National Council (CCNC) Toronto Chapter, reached 1,138 nationally at the time of writing (CCNC 2022). A survey on anti‐Asian racism collected and released by the CCNC and Project 1907 showed a 47% increase in incidents in 2021 compared to 2020 (Balintec 2022). Anti‐Asian racism has continued and/or increased in public and semi‐public spaces throughout the pandemic, highlighting how participation and mobility in the public sphere is privileged. While these data provide a clear indication of rising rates of racism in Canada during the pandemic (with increased rates across visible minority populations), unreported incidents and encounters make these numbers much higher.

Fear of hate incidents can affect the physical and mental health of racial minority groups and can limit participation in public space. In 2020, a Chinese Canadian teen was riding their bicycle in Saskatoon when a stranger began to shout racial slurs and accusations of spreading COVID‐19 before knocking them down and physically attacking them (*CBC* 2020). Writing from Vancouver, Liao (2020) highlights the effects of anti‐Asian racism on their movements through the city: “I now weigh the risk of a racist incident when deciding whether to go out for an errand….The resurgent racism has made my belonging here feel conditional.” Racialized people have always experienced racism and harassment in public space, but new reasons and methods have marked an escalation of incidents. Rising incidents, coupled with the emphasis placed on accessing public space for mental and physical health during the pandemic, speaks to the need for interventions (e.g., anti‐hate education campaigns, policy changes).

Instead, most provinces have taken a law enforcement approach to COVID‐19 public health orders, using coercive tickets and fines to control the population. Racism coupled with targeted policing has further limited participation for racialized and low‐income populations. Greater police presence to enforce changing rules is disproportionately felt and present in urban areas that had greater police presence prior to COVID‐19 (Florida et al. 2021). A report by the Canadian Civil Liberties Association (CCLA 2020, iv) on policing during the pandemic found that racialized persons and minorities have been unfairly treated by law enforcement during COVID‐19: “the arbitrary rules, increased enforcement powers, and significant fines are having a disproportionate impact on specific communities, including Black, Indigenous, and other racialized groups, those with precarious housing, recent immigrants, youth, members of the LGBTQ2S community, and certain religious minorities.” These communities are also the most likely to suffer from layoffs, to be visible in low‐wage essential service work, to rely on public transportation, to lose access to services, to lack the necessary private and/or public spaces to socially distance, and to experience the highest rates of transmission.

COVID‐19 has exacerbated the need for social and community supports, especially for vulnerable members of society. The homeless population is particularly at risk during the pandemic given challenges in accessing health services and supports: “People who are homeless would experience a differential in exposure, susceptibility, and access to treatment” (Baral et al. 2021, 929). To facilitate physical distancing and to meet public health guidelines, shelters were forced to cut back on their number of beds (*CBC Radio* 2020). This resulted in people being temporarily moved into hotels and the development of homeless encampments in public parks as many struggled to find safety. The response to these encampments by municipal governments is consistent: these are illegal settlements in violation of bylaws preventing sleeping overnight in public parks with the potential for violent removal (CCLA 2020). Clashes between homeless persons and police over bylaw infringement have taken place for decades. The pandemic has exacerbated the conditions of homelessness (e.g., job loss, mental and physical health, addictions) without adequate supports, making the (at times violent) removal of encampments a disturbing misuse of power.

As Mitchell (1995) suggests, public space is a space of and for control. It is clear that the pandemic has extenuated unequal access, participation, and belonging in public space. Extending police powers to apply vague public health orders has led to bias and discrimination towards racialized populations and minorities, with increased ticketing and fines reported (CCLA 2020). In addition, incidents of anti‐Asian and xenophobic racism and discrimination across the country have intensified, including hate crimes against children and elderly populations. We have taken a law enforcement approach to homelessness during a period where access to services and supports (and affordable housing) is lacking. People experiencing homelessness are not able to socially distance, self‐isolate or stay at home, placing them at greater risk of transmission. As a result, during COVID‐19 visible homelessness increased in urban centres (Olson and Pauly 2021). Punitive measures (fines and ticketing) associated with emergency health orders (consider Ontario's stay at home order or Quebec's curfew policy) disproportionately affect marginalized populations in public space, including persons experiencing homelessness (CCLA 2021). It is critical that municipalities commit to providing inclusive public spaces where *all* bodies can exist outside of the threat of violence.

## 4 The rise of individualized consumption and private space

In stark contrast to the injustices surrounding public space raised above (both unequal access and participation for racialized and marginalized populations), individualized consumption patterns have grown. The shift towards compartmentalized experiences of individualized space threaten the existence of, and investment into, shared public space. Despite isolation, the collective impulse to consume recreation and sport during COVID‐19 is palpable. Media reports of bike, recreational vehicle, swing set, and trampoline shortages in the spring/summer morphed into supply chain issues with skis and skates with the passing of another season. Campgrounds across provinces sold out early to meet the demand for staycations. These are all individualized uses of space: from skis to bikes to camp sites.

Connected to this, while the need for public space has risen sharply during the pandemic for physical and mental health and to accommodate social distancing, so too has demand for private space for those able to afford it. The sale of larger homes on larger tracts of land on the periphery/within commuting distance of urban centres, alongside increased demand for cottages and cabins, has defined the housing market during this period of crisis. The sale of cottages increased by an average of 15% nationally in 2020 and 2021 (Senoran 2021). The housing market similarly experienced heightened demand and inflated sale prices, raising issues surrounding affordability. The desire for greater amounts of space and decentralized living is not new. Canadian municipalities experienced the movement of middle to upper class people out of city centres starting in the late 19^th^ century, when urbanization and industrialization caused health issues (e.g., pollution, disease, overcrowding). This drive towards urban decentralization to acquire more private space is wreaking havoc on intensification planning, a directive that has long formed a critical component of municipal planning practices. Intensification planning focuses on mixed‐use central and compact development and emerged as a response to 20^th^‐century decentralization trends that emphasized automobile dependency and low‐density single land use developments (Graham et al. 2019).

In Regina, decades without limitations on outward growth resulted in peripheral expansion (Graham 2018) and a downtown that lacks vitality or population. Despite commitments to intensification planning, Regina's City Council recently voted to review density targets in new peripheral neighbourhoods to determine the place for exclusive large‐lot developments in demand during COVID‐19. If these targets are lowered, it will mark a significant departure from *Design Regina*, the official community plan which includes a goal of attracting 10,000 new residents in the downtown over a 25‐year period (City of Regina 2013). Since 2013, 74 people have moved into the downtown. Commenting on the current trends, Mayor Sandra Masters remarked: “So, if my math is correct … in order to achieve 10,000 at this current trend rate it would be 957 years to reach that target?” (Masters, quoted in Ackerman 2021). The continued expansion outward coupled with the need to service decentralized developments outside of the core will lessen funding and investment into shared resources in the inner city where low‐income neighbourhoods are concentrated.

Municipalities need to exercise caution if departing from long‐term planning to meet current demand for private space. Shortchanging long‐term planning to meet short‐term demand, in tandem with discussions surrounding the privatization of parks (Kline 2021), is likely to exacerbate unequal access to (public) space. Demand for individualized experiences of public space, and increased private space, must be read through the lens of privilege. Planning practices and policies must be particularly monitored across urban centres during COVID‐19, to ensure compliance with long‐term development plans that emphasize compact development, shared resources, and investments into inner city communities.

## 5 Conclusion: The future of public space

This viewpoint highlights the importance of constructing inclusive public spaces that are adaptable in the midst of crisis, and the protection of those that already exist as spaces of refuge across Canadian urban centres. It emphasizes the need to retain and build public space in high‐density, low‐income areas which are the most likely to experience overcrowding and which lack access to private space. There is an urgency surrounding access to housing and social supports for the most vulnerable members of our society, and a need to shift away from punitive policing measures for social control. Shifts in planning practices in local settings must be monitored during the crisis to ensure that they are not departing from long‐term goals for intensification and shared resources, in order to satisfy short‐term demands for private space. On the one hand, the COVID‐19 pandemic has illustrated the need for public space to support social distancing and physical and mental health; on the other hand, it has deepened the inequities that have long existed in cities. It has exposed the dismantling and lack of funding to support social services and affordability, and the poor living conditions that many have to endure. It has given rise to increased racial discrimination and harassment by law enforcement, through the unequal application of vague health orders, and by the white population, who blame racialized populations for the spread of disease.

While I have referred to some of Canada's largest urban centres in this viewpoint, we need to be careful not to generalize the trends that we are seeing in large urban centres as applicable down the urban hierarchy. Mid‐sized peripheral cities and smaller cities are affected differently than large metropolitan areas. We are mostly hearing about larger cities in mainstream media, but as geographers we have a role to play in ensuring that alternative stories and experiences are also told.

COVID‐19 transformed how we view space and how we embody space. It has shaped how we want to live and where we want to live. It has deepened the inequities that characterize our urban centres, with new sets of skis and cottages for some and a loss of livelihood, safety, and shelter for others. It has exposed the contradictions of public space as a site of unpredictability and as a site for control and order. As March and Lehrer (2021, 16) suggest, “public space cannot be a privileged domain of planners and policy makers, something physical we put out there to be used or consumed but is instead something we all participate in generating and therefore are collectively responsible for”. If we take seriously this idea of public space as a collective responsibility, as something which is constantly being produced and reproduced, now is the time to advocate for public spaces where everyone has the right to exist.

## 6 Acknowledgements

Open Access for this article was supported by the President's Publication Fund and Arts Publication Fund at the University of Regina.
